# Supplementary figures and images for: Coupling of autism genes to tissue-wide expression and dysfunction of synapse, calcium signalling and transcriptional regulation
Source: PLoS One. 2020 Dec 18;15(12):e0242773. doi: 10.1371/journal.pone.0242773 (PMC7748153; doi:10.1371/journal.pone.0242773)

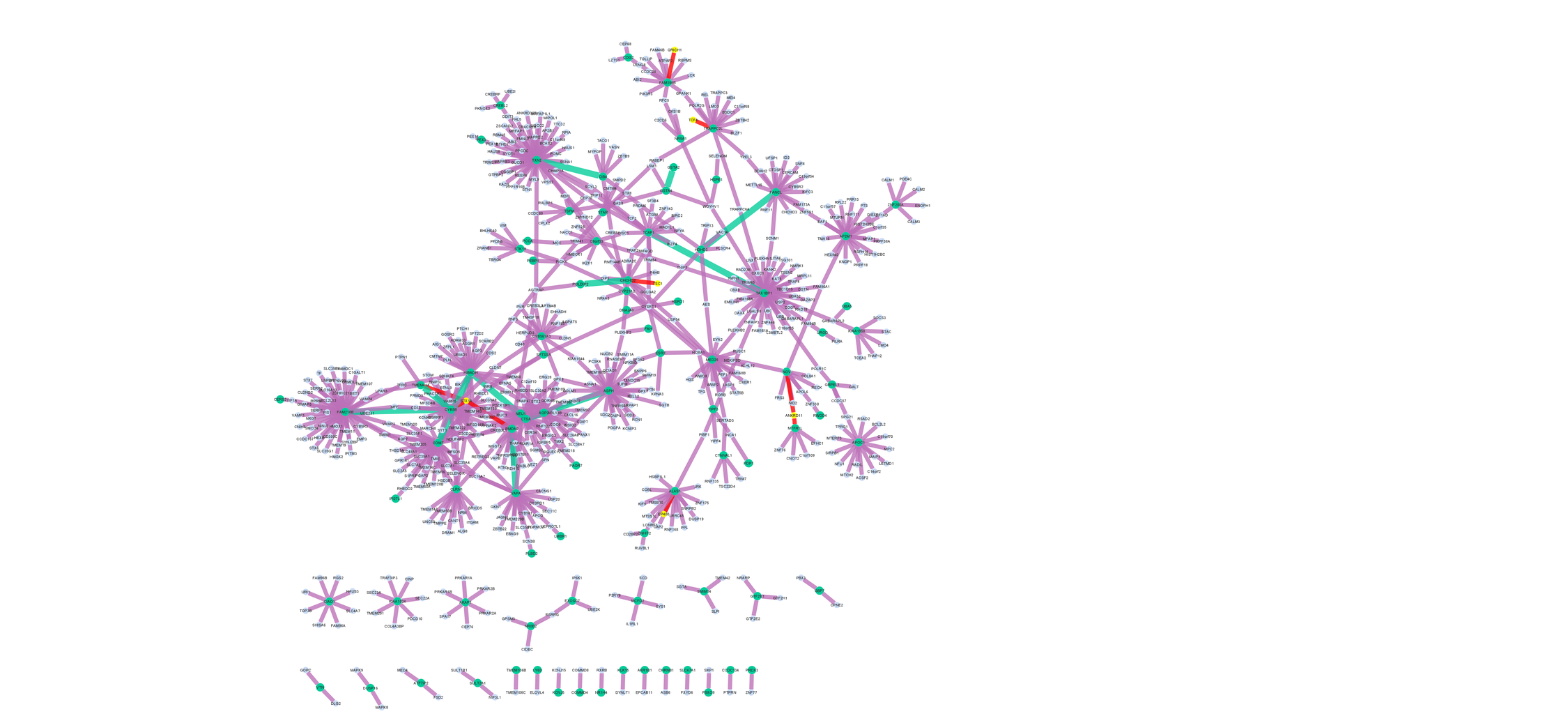

Supplement: S1 Fig — Yellow nodes highlight ASD genes, green are tissue-specific genes, red edges are interactions between ASD genes and other partners. The graphs are in order of appearance; Adrenal Gland, Adipose subcutaneous, Artery aorta, Artery coronary, Artery tibial, Colon sigmoid, Colon transverse, Esophagus-mucosa, Esophagus-muscularis, Heart-left atrial appendage, Heart-left ventricle, Kidney, Lung, Muscle-skeletal, Pancreas, Pituitary, Small Intestine-terminal ileum, Spleen, Stomach. (TIF) [file pone.0242773.s001.tif]

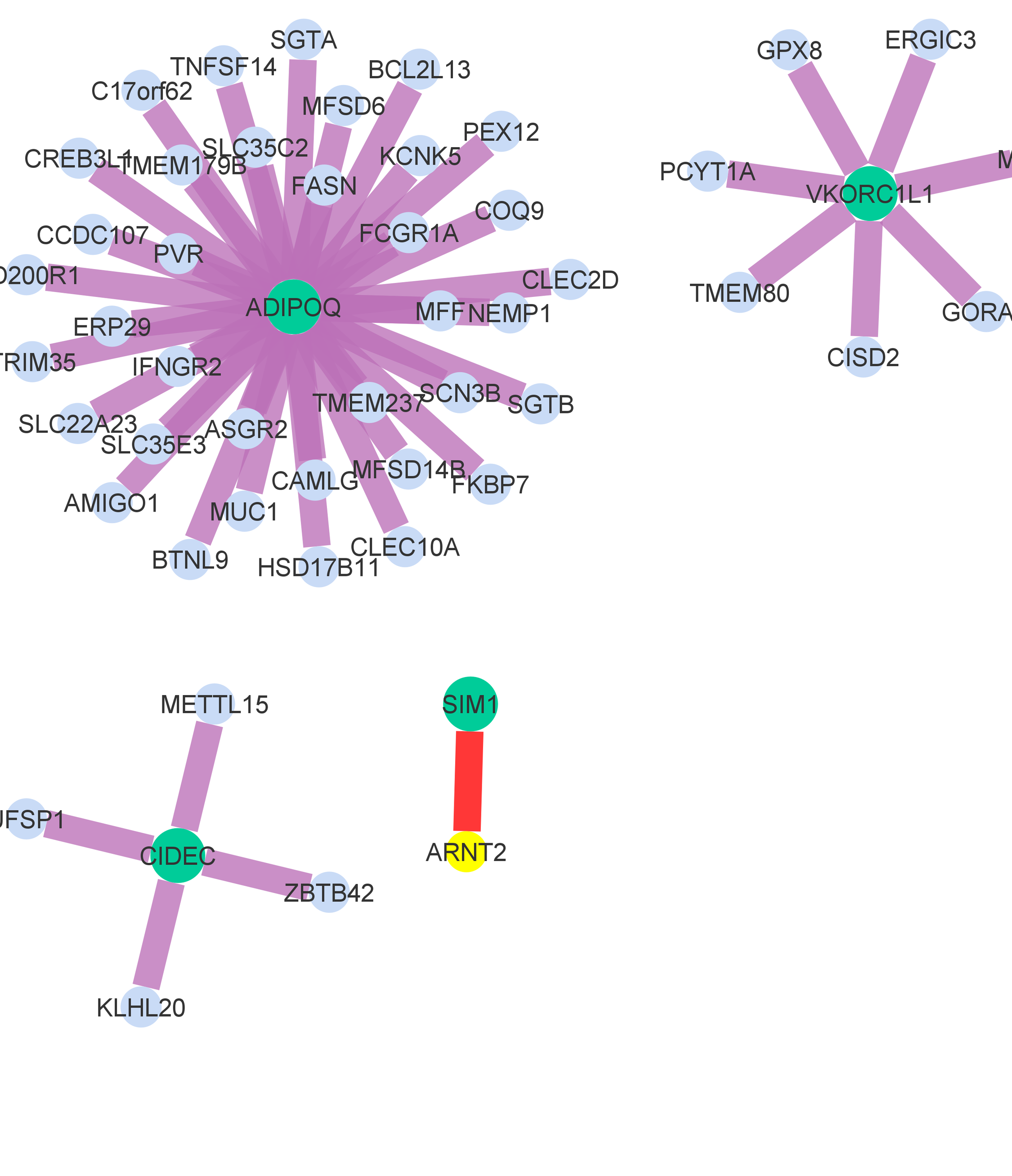

Supplement: S2 Fig — Yellow nodes highlight ASD genes, green are tissue-specific genes, red edges are interactions between ASD genes and other partners. The graphs are in order of appearance; Adrenal Gland, Adipose subcutaneous, Artery aorta, Artery coronary, Artery tibial, Colon sigmoid, Colon transverse, Esophagus-mucosa, Esophagus-muscularis, Heart-left atrial appendage, Heart-left ventricle, Kidney, Lung, Muscle-skeletal, Pancreas, Pituitary, Small Intestine-terminal ileum, Spleen, Stomach. (TIF) [file pone.0242773.s002.tif]

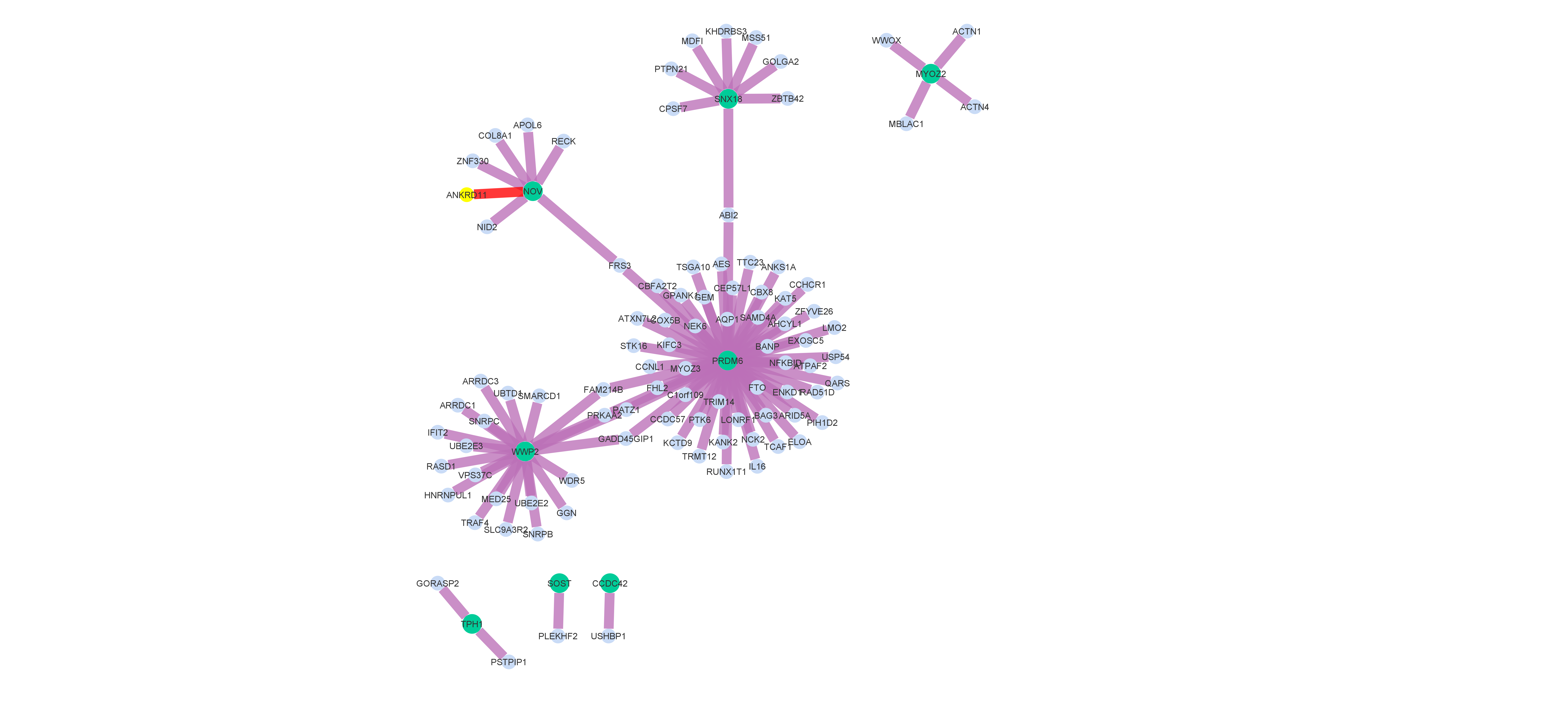

Supplement: S3 Fig — Yellow nodes highlight ASD genes, green are tissue-specific genes, red edges are interactions between ASD genes and other partners. The graphs are in order of appearance; Adrenal Gland, Adipose subcutaneous, Artery aorta, Artery coronary, Artery tibial, Colon sigmoid, Colon transverse, Esophagus-mucosa, Esophagus-muscularis, Heart-left atrial appendage, Heart-left ventricle, Kidney, Lung, Muscle-skeletal, Pancreas, Pituitary, Small Intestine-terminal ileum, Spleen, Stomach. (PNG) [file pone.0242773.s003.png]

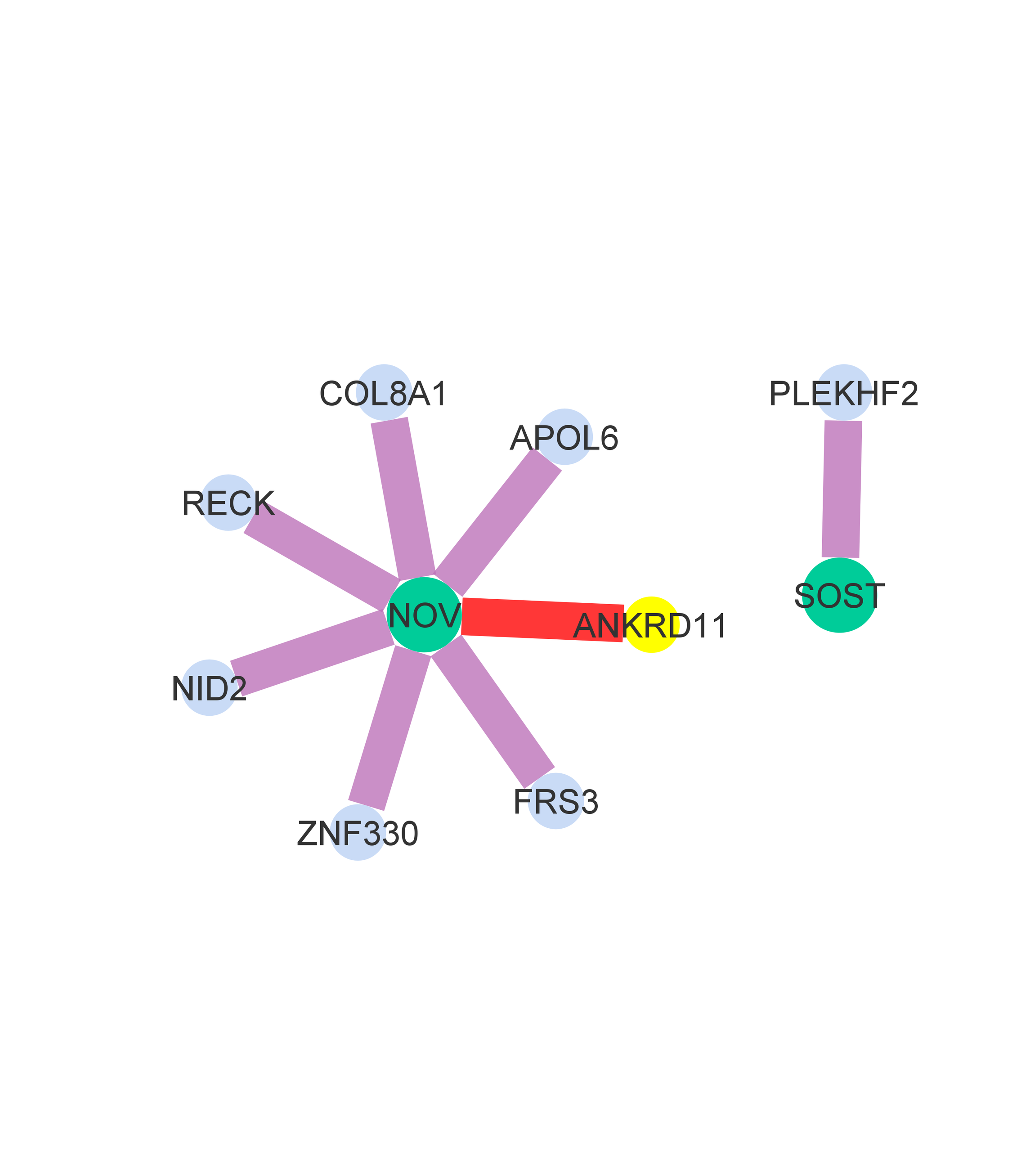

Supplement: S4 Fig — Yellow nodes highlight ASD genes, green are tissue-specific genes, red edges are interactions between ASD genes and other partners. The graphs are in order of appearance; Adrenal Gland, Adipose subcutaneous, Artery aorta, Artery coronary, Artery tibial, Colon sigmoid, Colon transverse, Esophagus-mucosa, Esophagus-muscularis, Heart-left atrial appendage, Heart-left ventricle, Kidney, Lung, Muscle-skeletal, Pancreas, Pituitary, Small Intestine-terminal ileum, Spleen, Stomach. (TIF) [file pone.0242773.s004.tif]

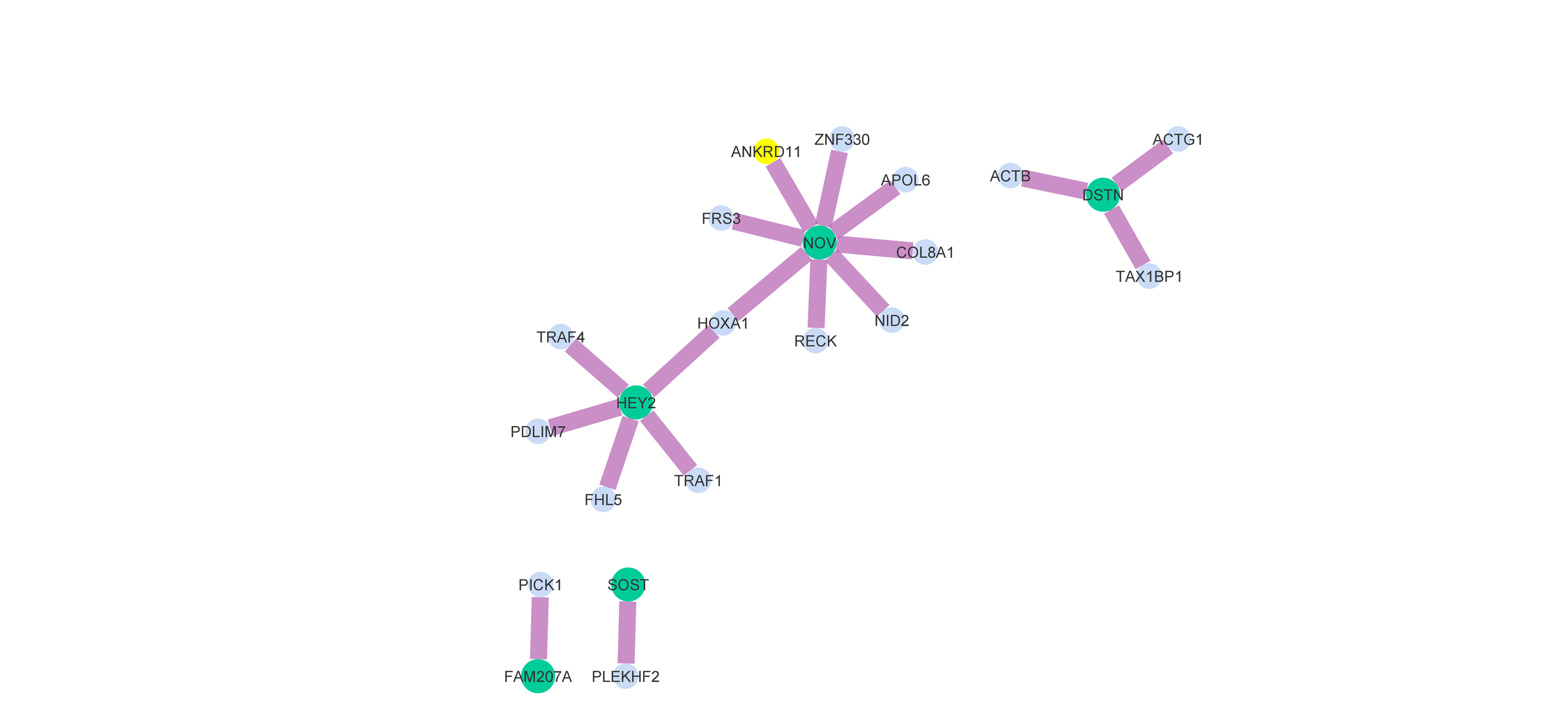

Supplement: S5 Fig — Yellow nodes highlight ASD genes, green are tissue-specific genes, red edges are interactions between ASD genes and other partners. The graphs are in order of appearance; Adrenal Gland, Adipose subcutaneous, Artery aorta, Artery coronary, Artery tibial, Colon sigmoid, Colon transverse, Esophagus-mucosa, Esophagus-muscularis, Heart-left atrial appendage, Heart-left ventricle, Kidney, Lung, Muscle-skeletal, Pancreas, Pituitary, Small Intestine-terminal ileum, Spleen, Stomach. (TIF) [file pone.0242773.s005.tif]

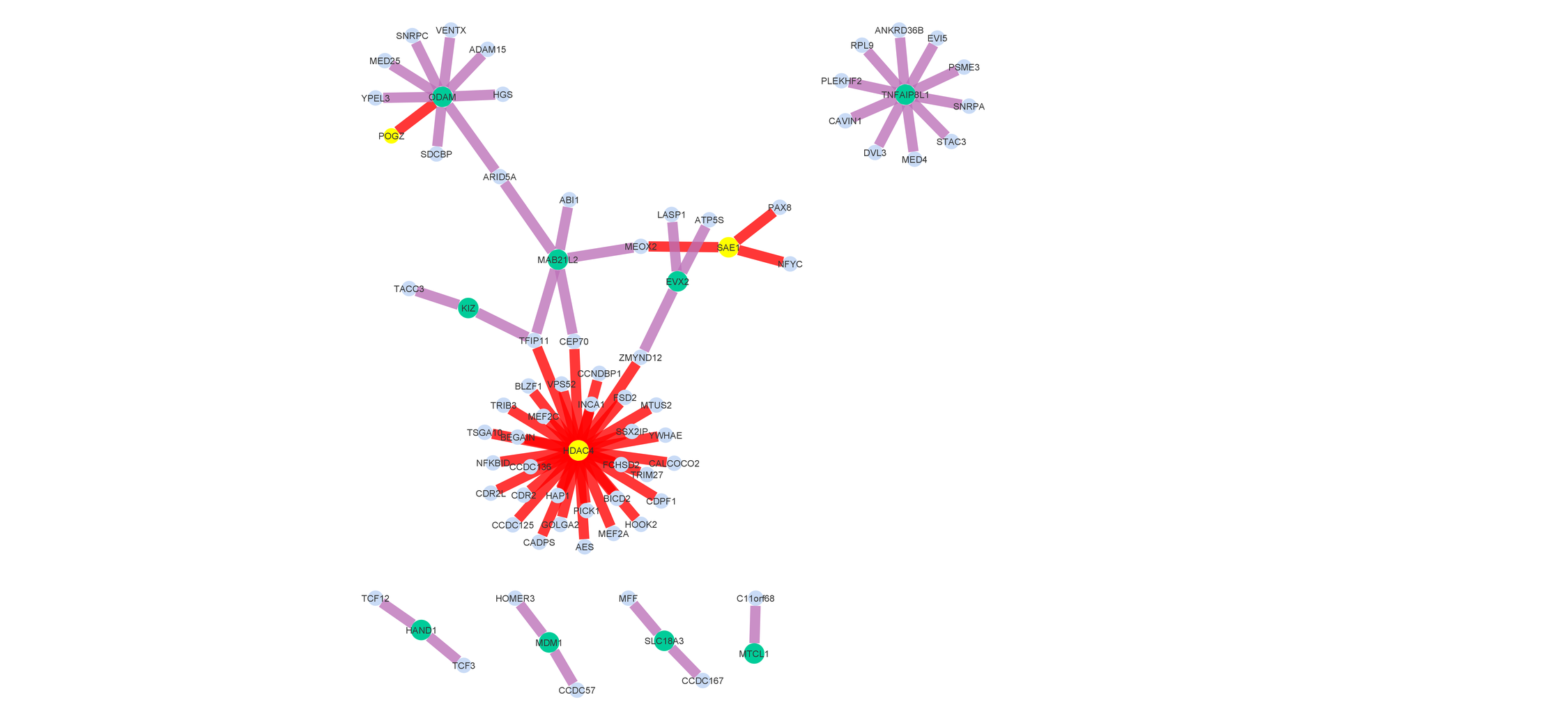

Supplement: S6 Fig — Yellow nodes highlight ASD genes, green are tissue-specific genes, red edges are interactions between ASD genes and other partners. The graphs are in order of appearance; Adrenal Gland, Adipose subcutaneous, Artery aorta, Artery coronary, Artery tibial, Colon sigmoid, Colon transverse, Esophagus-mucosa, Esophagus-muscularis, Heart-left atrial appendage, Heart-left ventricle, Kidney, Lung, Muscle-skeletal, Pancreas, Pituitary, Small Intestine-terminal ileum, Spleen, Stomach. (TIF) [file pone.0242773.s006.tif]

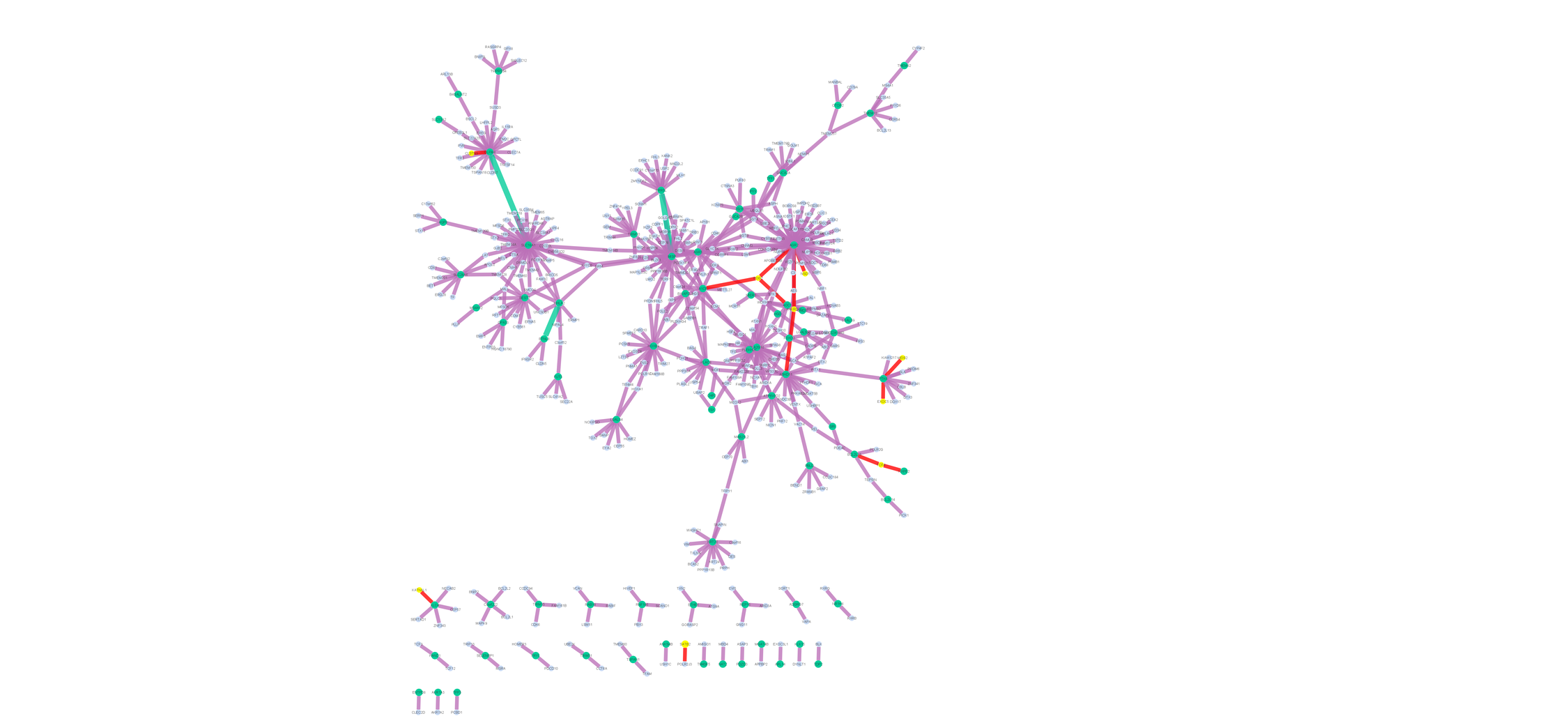

Supplement: S7 Fig — Yellow nodes highlight ASD genes, green are tissue-specific genes, red edges are interactions between ASD genes and other partners. The graphs are in order of appearance; Adrenal Gland, Adipose subcutaneous, Artery aorta, Artery coronary, Artery tibial, Colon sigmoid, Colon transverse, Esophagus-mucosa, Esophagus-muscularis, Heart-left atrial appendage, Heart-left ventricle, Kidney, Lung, Muscle-skeletal, Pancreas, Pituitary, Small Intestine-terminal ileum, Spleen, Stomach. (TIF) [file pone.0242773.s007.tif]

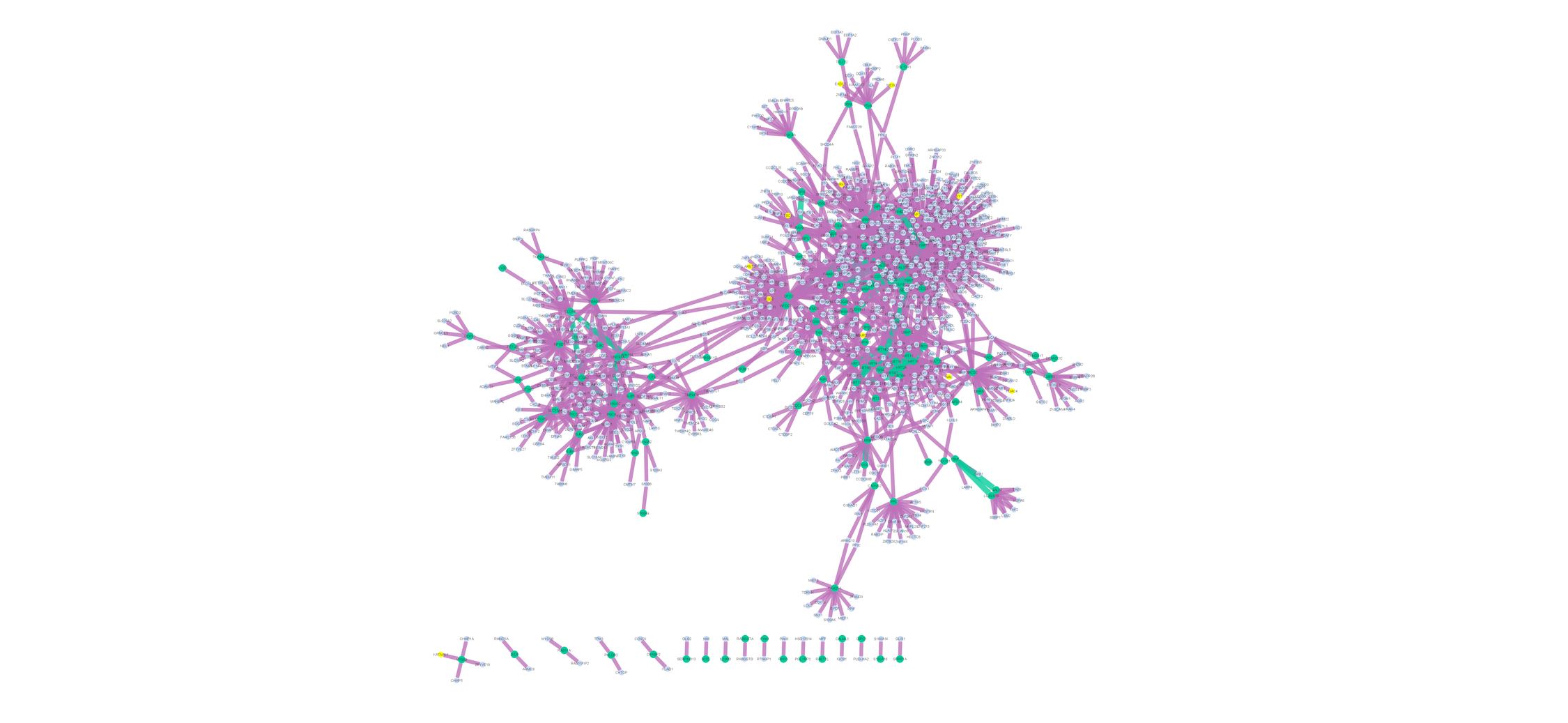

Supplement: S8 Fig — Yellow nodes highlight ASD genes, green are tissue-specific genes, red edges are interactions between ASD genes and other partners. The graphs are in order of appearance; Adrenal Gland, Adipose subcutaneous, Artery aorta, Artery coronary, Artery tibial, Colon sigmoid, Colon transverse, Esophagus-mucosa, Esophagus-muscularis, Heart-left atrial appendage, Heart-left ventricle, Kidney, Lung, Muscle-skeletal, Pancreas, Pituitary, Small Intestine-terminal ileum, Spleen, Stomach. (TIF) [file pone.0242773.s008.tif]

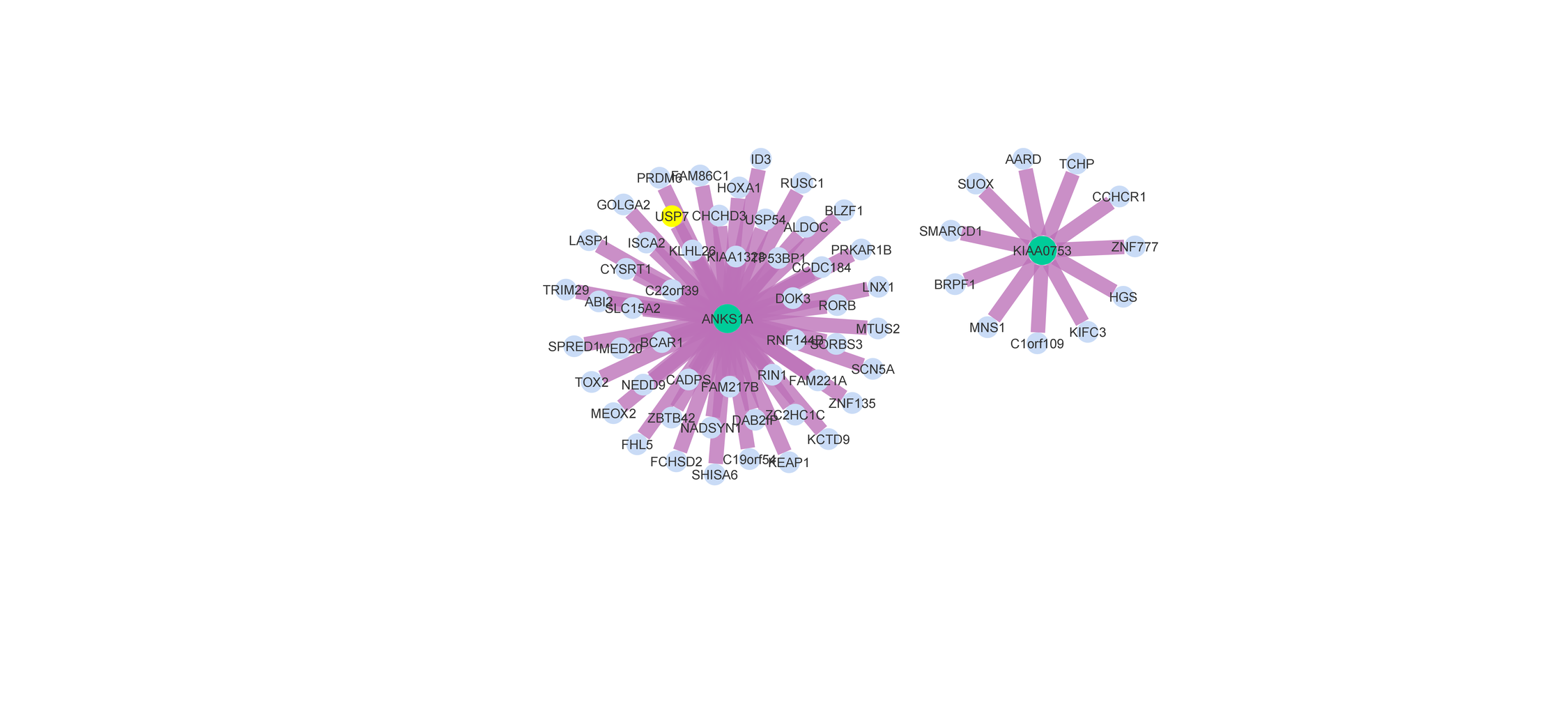

Supplement: S9 Fig — Yellow nodes highlight ASD genes, green are tissue-specific genes, red edges are interactions between ASD genes and other partners. The graphs are in order of appearance; Adrenal Gland, Adipose subcutaneous, Artery aorta, Artery coronary, Artery tibial, Colon sigmoid, Colon transverse, Esophagus-mucosa, Esophagus-muscularis, Heart-left atrial appendage, Heart-left ventricle, Kidney, Lung, Muscle-skeletal, Pancreas, Pituitary, Small Intestine-terminal ileum, Spleen, Stomach. (TIF) [file pone.0242773.s009.tif]

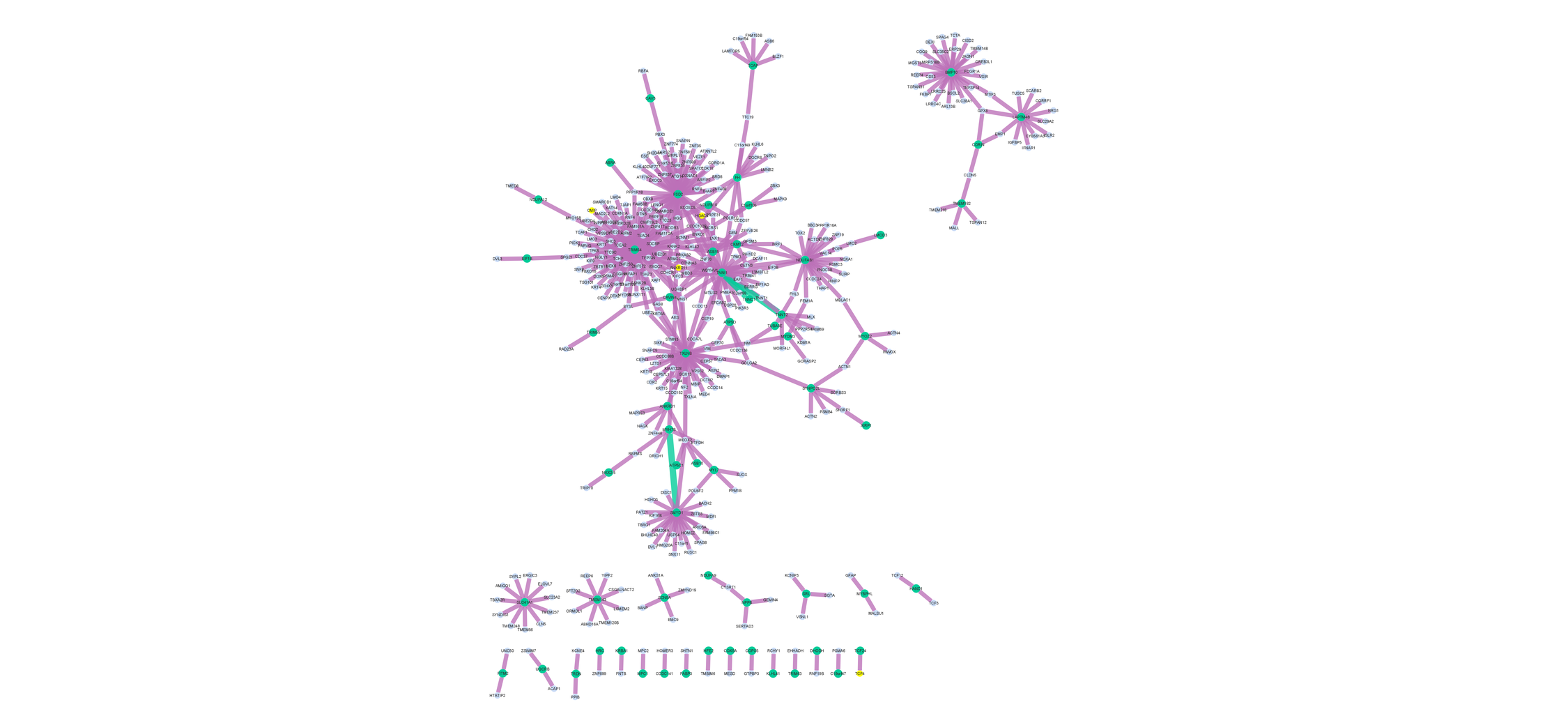

Supplement: S10 Fig — Yellow nodes highlight ASD genes, green are tissue-specific genes, red edges are interactions between ASD genes and other partners. The graphs are in order of appearance; Adrenal Gland, Adipose subcutaneous, Artery aorta, Artery coronary, Artery tibial, Colon sigmoid, Colon transverse, Esophagus-mucosa, Esophagus-muscularis, Heart-left atrial appendage, Heart-left ventricle, Kidney, Lung, Muscle-skeletal, Pancreas, Pituitary, Small Intestine-terminal ileum, Spleen, Stomach. (TIF) [file pone.0242773.s010.tif]

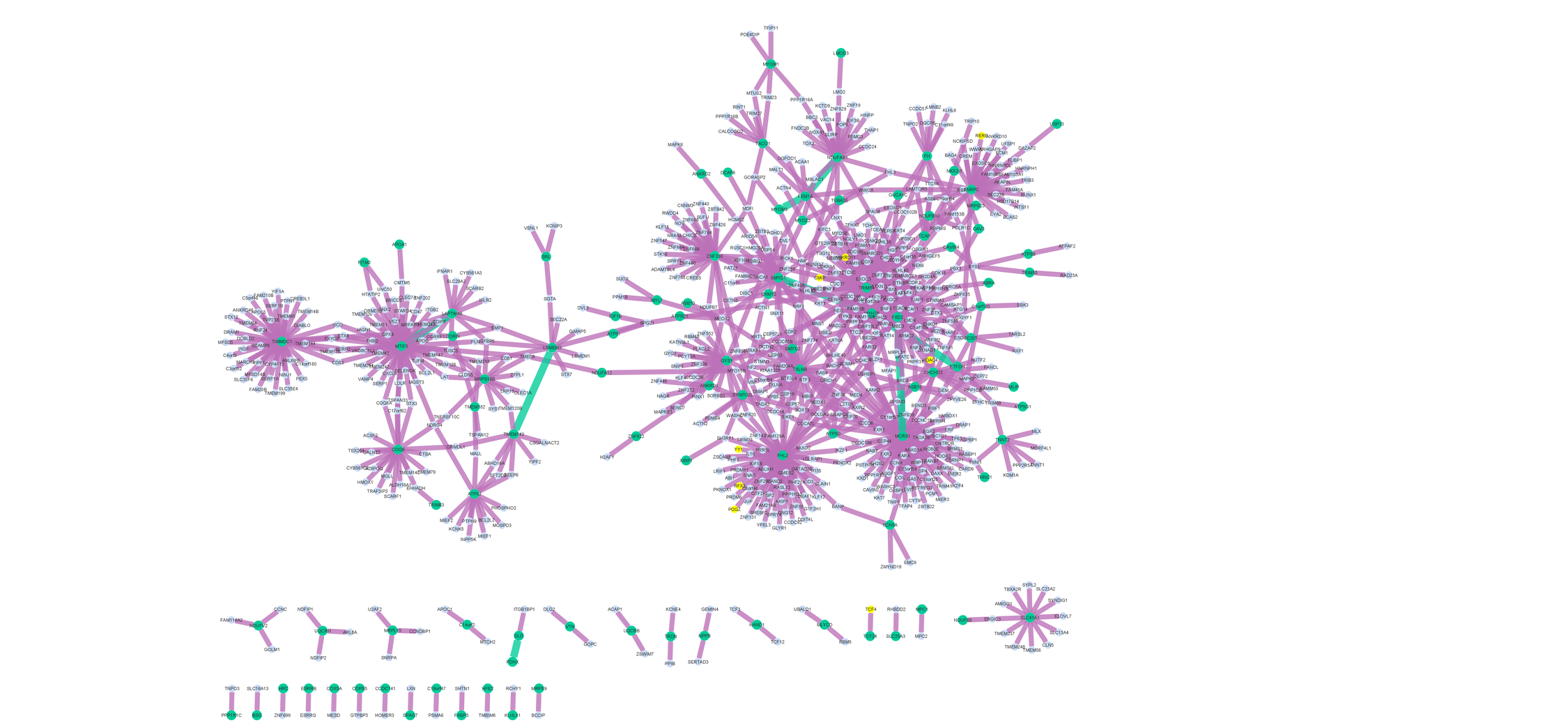

Supplement: S11 Fig — Yellow nodes highlight ASD genes, green are tissue-specific genes, red edges are interactions between ASD genes and other partners. The graphs are in order of appearance; Adrenal Gland, Adipose subcutaneous, Artery aorta, Artery coronary, Artery tibial, Colon sigmoid, Colon transverse, Esophagus-mucosa, Esophagus-muscularis, Heart-left atrial appendage, Heart-left ventricle, Kidney, Lung, Muscle-skeletal, Pancreas, Pituitary, Small Intestine-terminal ileum, Spleen, Stomach. (TIF) [file pone.0242773.s011.tif]

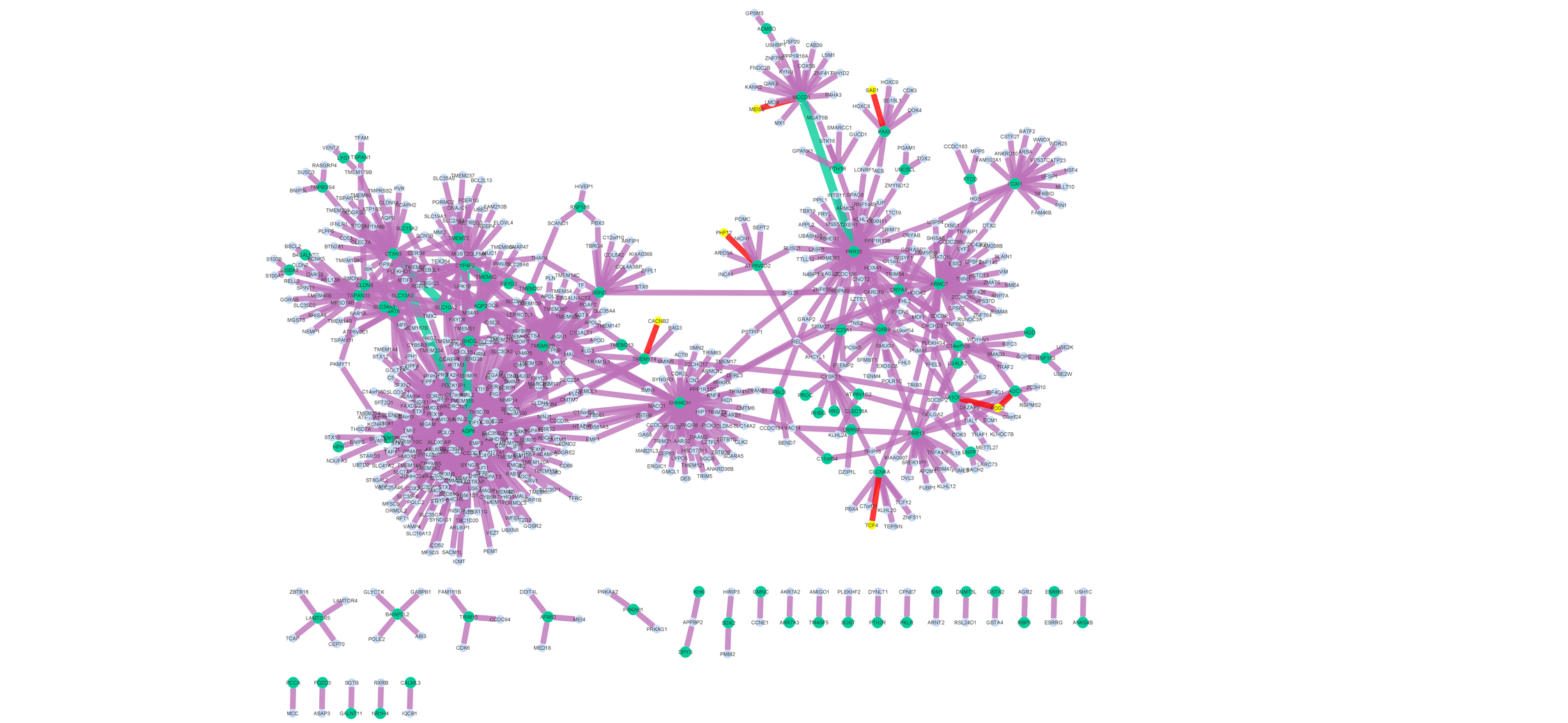

Supplement: S12 Fig — Yellow nodes highlight ASD genes, green are tissue-specific genes, red edges are interactions between ASD genes and other partners. The graphs are in order of appearance; Adrenal Gland, Adipose subcutaneous, Artery aorta, Artery coronary, Artery tibial, Colon sigmoid, Colon transverse, Esophagus-mucosa, Esophagus-muscularis, Heart-left atrial appendage, Heart-left ventricle, Kidney, Lung, Muscle-skeletal, Pancreas, Pituitary, Small Intestine-terminal ileum, Spleen, Stomach. (TIF) [file pone.0242773.s012.tif]

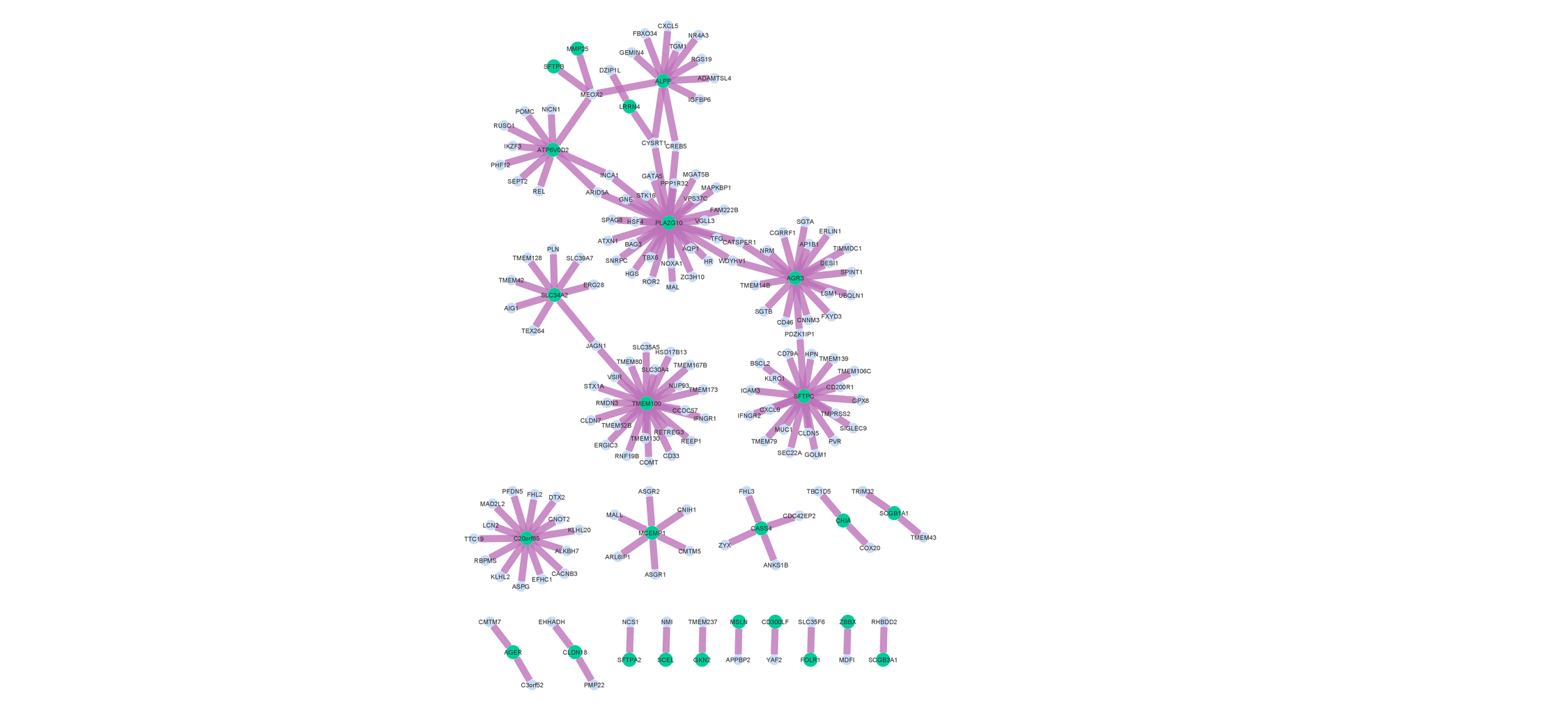

Supplement: S13 Fig — Yellow nodes highlight ASD genes, green are tissue-specific genes, red edges are interactions between ASD genes and other partners. The graphs are in order of appearance; Adrenal Gland, Adipose subcutaneous, Artery aorta, Artery coronary, Artery tibial, Colon sigmoid, Colon transverse, Esophagus-mucosa, Esophagus-muscularis, Heart-left atrial appendage, Heart-left ventricle, Kidney, Lung, Muscle-skeletal, Pancreas, Pituitary, Small Intestine-terminal ileum, Spleen, Stomach. (TIF) [file pone.0242773.s013.tif]

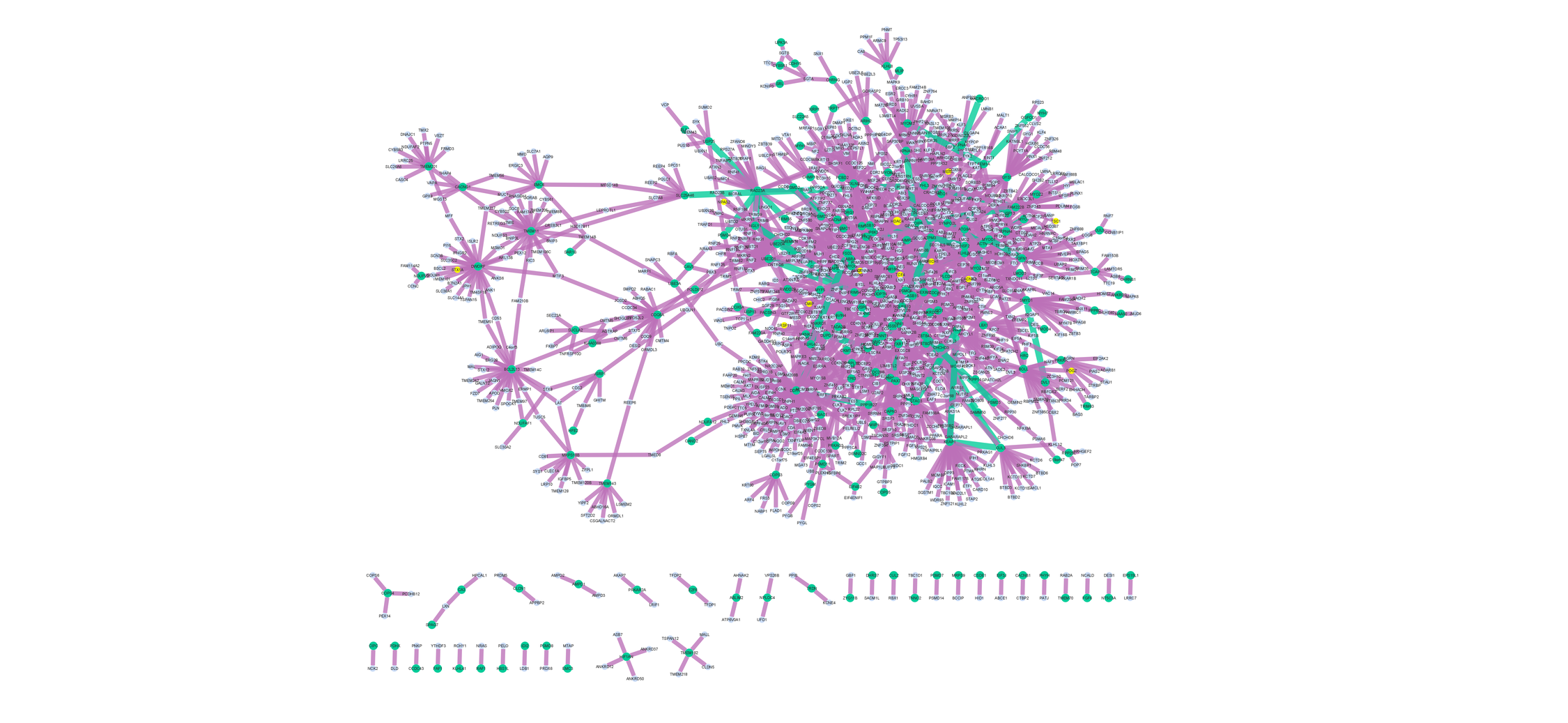

Supplement: S14 Fig — Yellow nodes highlight ASD genes, green are tissue-specific genes, red edges are interactions between ASD genes and other partners. The graphs are in order of appearance; Adrenal Gland, Adipose subcutaneous, Artery aorta, Artery coronary, Artery tibial, Colon sigmoid, Colon transverse, Esophagus-mucosa, Esophagus-muscularis, Heart-left atrial appendage, Heart-left ventricle, Kidney, Lung, Muscle-skeletal, Pancreas, Pituitary, Small Intestine-terminal ileum, Spleen, Stomach. (TIF) [file pone.0242773.s014.tif]

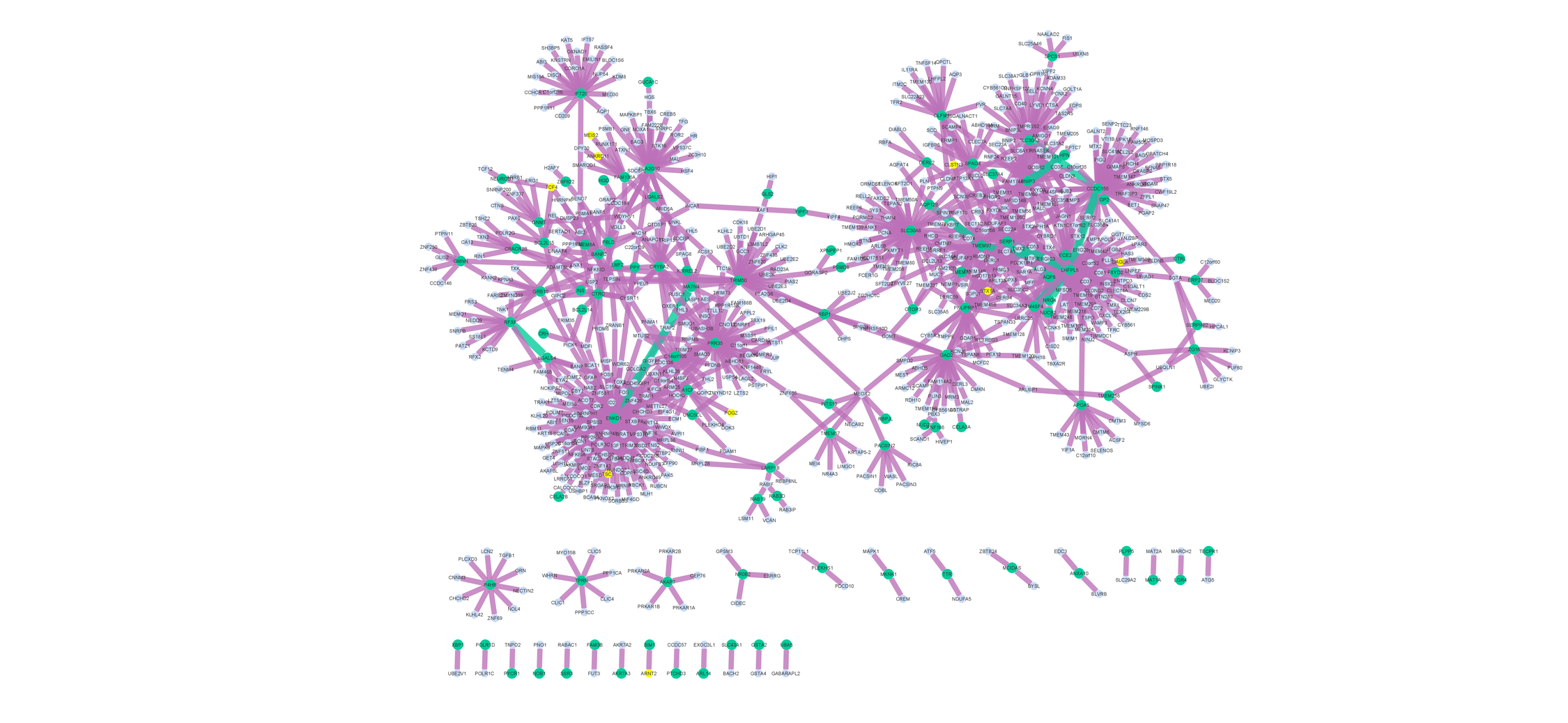

Supplement: S15 Fig — Yellow nodes highlight ASD genes, green are tissue-specific genes, red edges are interactions between ASD genes and other partners. The graphs are in order of appearance; Adrenal Gland, Adipose subcutaneous, Artery aorta, Artery coronary, Artery tibial, Colon sigmoid, Colon transverse, Esophagus-mucosa, Esophagus-muscularis, Heart-left atrial appendage, Heart-left ventricle, Kidney, Lung, Muscle-skeletal, Pancreas, Pituitary, Small Intestine-terminal ileum, Spleen, Stomach. (TIF) [file pone.0242773.s015.tif]

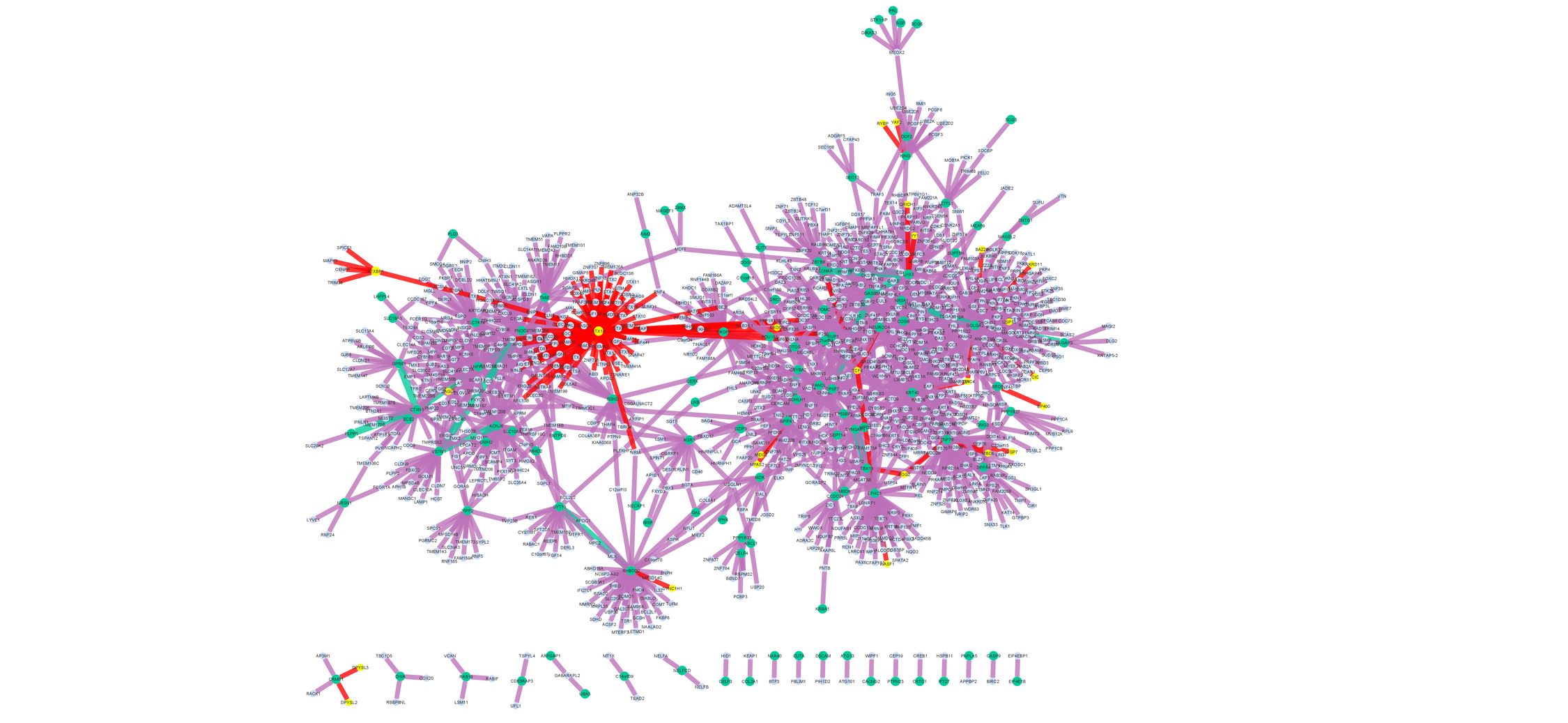

Supplement: S16 Fig — Yellow nodes highlight ASD genes, green are tissue-specific genes, red edges are interactions between ASD genes and other partners. The graphs are in order of appearance; Adrenal Gland, Adipose subcutaneous, Artery aorta, Artery coronary, Artery tibial, Colon sigmoid, Colon transverse, Esophagus-mucosa, Esophagus-muscularis, Heart-left atrial appendage, Heart-left ventricle, Kidney, Lung, Muscle-skeletal, Pancreas, Pituitary, Small Intestine-terminal ileum, Spleen, Stomach. (TIF) [file pone.0242773.s016.tif]

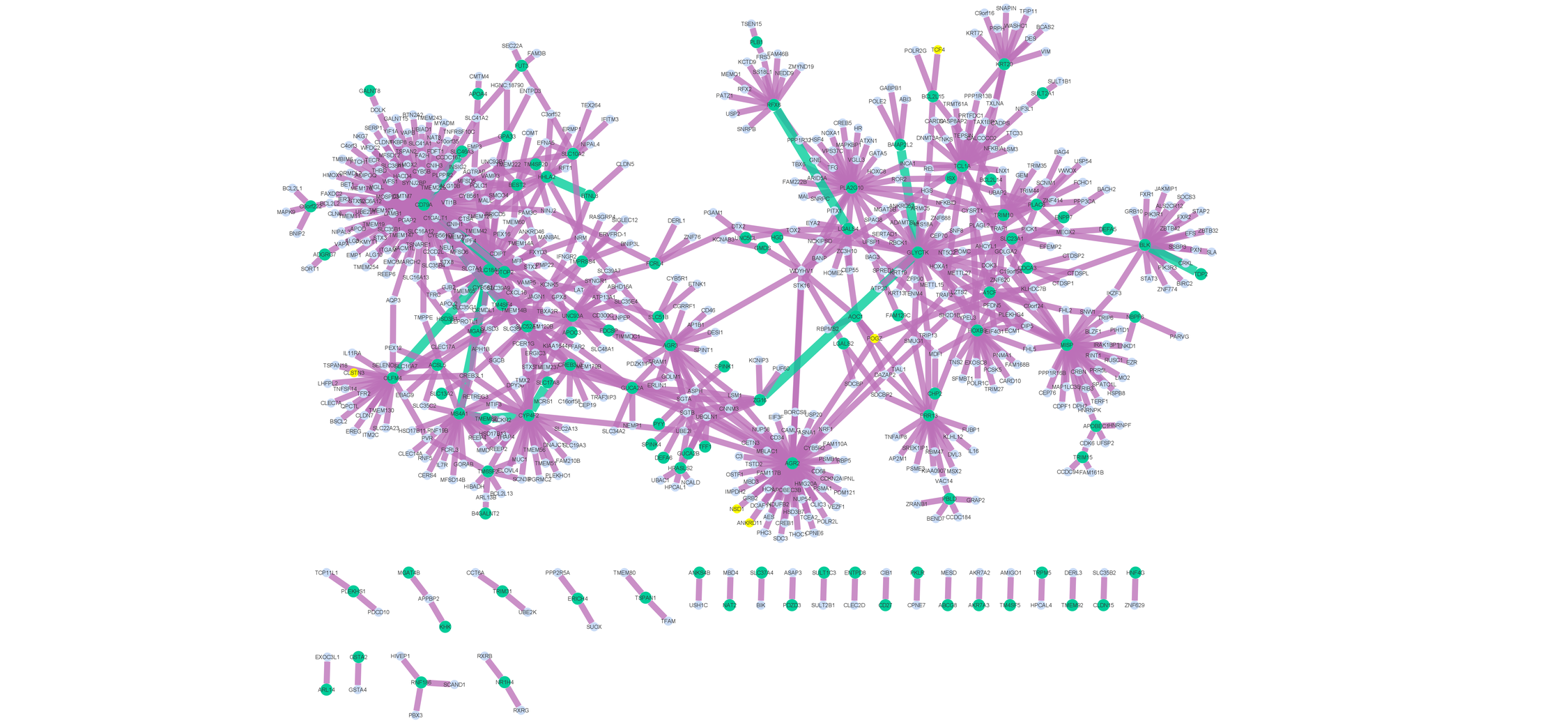

Supplement: S17 Fig — Yellow nodes highlight ASD genes, green are tissue-specific genes, red edges are interactions between ASD genes and other partners. The graphs are in order of appearance; Adrenal Gland, Adipose subcutaneous, Artery aorta, Artery coronary, Artery tibial, Colon sigmoid, Colon transverse, Esophagus-mucosa, Esophagus-muscularis, Heart-left atrial appendage, Heart-left ventricle, Kidney, Lung, Muscle-skeletal, Pancreas, Pituitary, Small Intestine-terminal ileum, Spleen, Stomach. (TIF) [file pone.0242773.s017.tif]

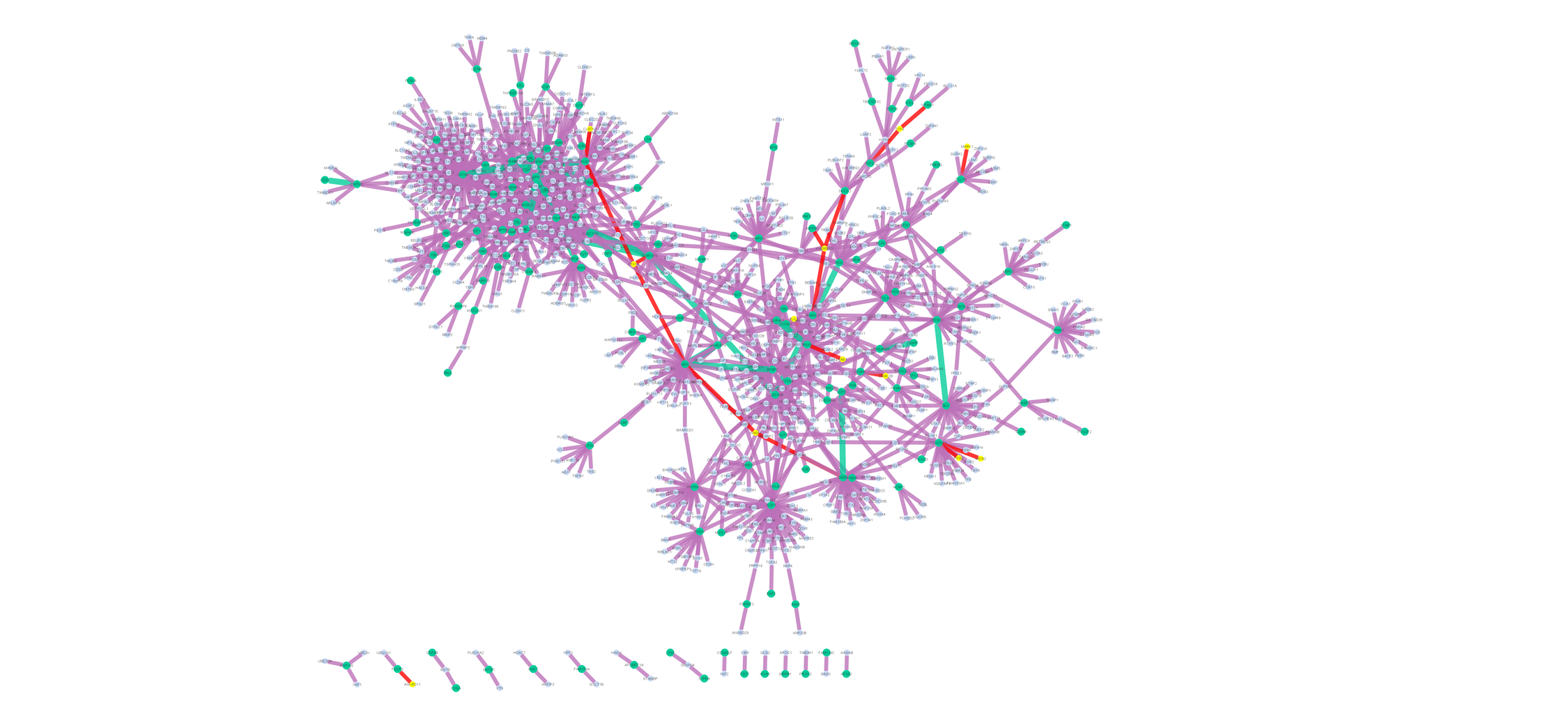

Supplement: S18 Fig — Yellow nodes highlight ASD genes, green are tissue-specific genes, red edges are interactions between ASD genes and other partners. The graphs are in order of appearance; Adrenal Gland, Adipose subcutaneous, Artery aorta, Artery coronary, Artery tibial, Colon sigmoid, Colon transverse, Esophagus-mucosa, Esophagus-muscularis, Heart-left atrial appendage, Heart-left ventricle, Kidney, Lung, Muscle-skeletal, Pancreas, Pituitary, Small Intestine-terminal ileum, Spleen, Stomach. (TIF) [file pone.0242773.s018.tif]

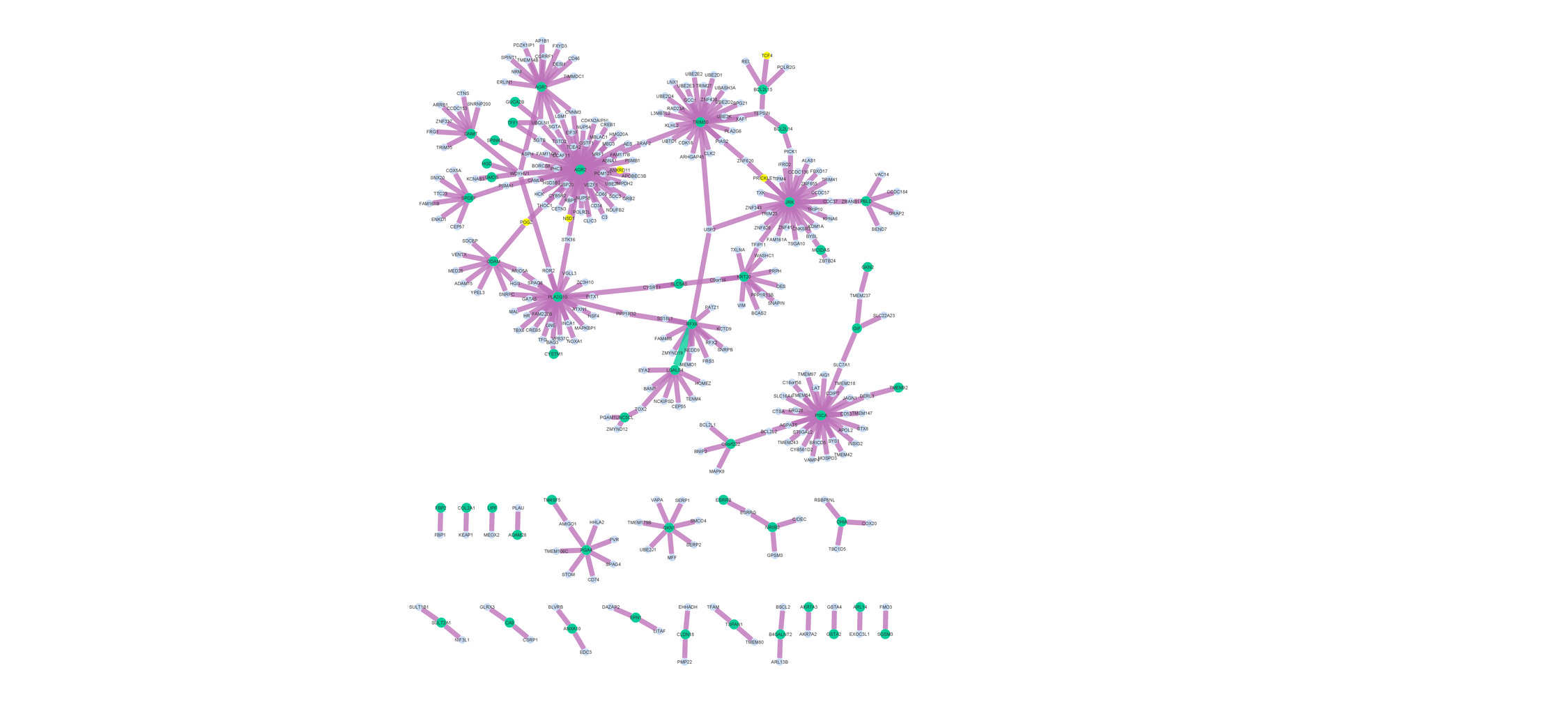

Supplement: S19 Fig — Yellow nodes highlight ASD genes, green are tissue-specific genes, red edges are interactions between ASD genes and other partners. The graphs are in order of appearance; Adrenal Gland, Adipose subcutaneous, Artery aorta, Artery coronary, Artery tibial, Colon sigmoid, Colon transverse, Esophagus-mucosa, Esophagus-muscularis, Heart-left atrial appendage, Heart-left ventricle, Kidney, Lung, Muscle-skeletal, Pancreas, Pituitary, Small Intestine-terminal ileum, Spleen, Stomach. (TIF) [file pone.0242773.s019.tif]
