# Supplementary material for: Coupling of autism genes to tissue-wide expression and dysfunction of synapse, calcium signalling and transcriptional regulation
Source: PLoS One. 2020 Dec 18;15(12):e0242773. doi: 10.1371/journal.pone.0242773 (PMC7748153; doi:10.1371/journal.pone.0242773)
Supplement: Attachment — Submitted filename: Reviewer questions and responses (1).docx [file pone.0242773.s049.docx]

Dear Editors,

You thank you and reviewers for kind remarks and constructive comments. We have addressed the points raised by the reviewers, with point-by-point response below. We have revised the manuscript with additional data which significantly strengthen the manuscript. We hope the revised manuscript can now be accepted for publication on PLOS One.

Kind regards

Reviewer 1 questions

1) It would further strengthen the analysis if the authors provide a comparison of expression profile of the genes analyzed in normal vs ASD patients. The authors could provide the comparison for whole or a subset of genes based on availability of data. This would help in narrowing down the focus on genes whose expressions vary in diseased state for future experimental studies.

Response : We thank Reviewer 1 for raising this good point. We have now added a table and data on genes that were reported to be differentially expressed in various studies that overlap with our geneset.

2) ASD is more prevalent in males compared to females and is also characterized by an early onset with respect to age of an individual. So, it would again strengthen the manuscript if the authors analyze expression pattern in males vs. females and whether there is any difference with age. In case there is a notable difference, then the data should be presented whereas if there is no skewed behavior, that need to be mentioned appropriately in the results.

Response: We have used gene lists reported by Shi et al 2016 and identified some of our ASD genes present across brain regions at prenatal stages with sex-bias expression (Fig. 8A and B). We also used CSEA webtool and found our geneset is enriched across various developmental stages in the human brain (Fig 8D). We also used GeneOveralp in Bioconductor to test significance of overlap in male biased and female biased prenatal gene sets, and identified 34 genes which are differentially spliced in male and female (Fig 8C).

Reviewer 2 questions

1) At present, there does not seem to be a lack of candidate genes for ASD. In the present form the manuscript identifies yet another candidate set, which does not illuminate what makes ASD distinct from other neurologic diseases, or whether ASD is a set of conditions. The manuscript would benefit from an informative comparison of the author's set with other diseases, identifying unique features of ASD.

Response: We have compared ASD geneset with genes from psychiatric conditions (via Psychgenet) and 4 peripheral conditions (Inflammatory bowel disease, arrythmia, Type 1 and Type 2 diabetes) from Harmonizome database (GWAS lists are from GWASdb dataset. We have identified subsets of genes overlapping with CNS snf peripheral conditions, as well as ASD unique genes (Fig. 9)

2) As the authors indicate, it is likely that the ASD-driving dysregulation(s) occur early in development and thus, the utility of the present set is unclear.

Response: We have used CSEA to test for enrichment of the ASD geneset in various brain regions across different timepoints of brain development and showed a high enrichment to specific regions, cortex in particular (Fig. 8D)
